# Editorial: 3D tissue models in infection research

**DOI:** 10.3389/fcimb.2022.969132

**Published:** 2022-07-22

**Authors:** Vera Kozjak-Pavlovic, Wenxia Song, Sina Bartfeld, Marco Metzger

**Affiliations:** ^1^ Department of Microbiology, Julius Maximilian University of Würzburg, Würzburg, Germany; ^2^ Department of Cell Biology and Molecular Genetics, University of Maryland, College Park, College Park, Maryland, United States; ^3^ Technical University of Berlin, Berlin, Berlin, Germany; ^4^ Fraunhofer Translational Center Regenerative Therapies, Fraunhofer Society (FHG), Munich, Bavaria, Germany

**Keywords:** 3D tissue, biomimetic models, tissue culture, infection biology, pathogen-host interaction

As the COVID19 pandemic has taught us, research on the interactions between pathogenic microorganisms and their hosts is vital not only for advancing our understanding of the underlying biological processes but also for the development of therapeutic strategies and vaccines. Since its beginnings, infection research has relied on animal or cell culture models, but recently a plethora of new model systems have been established with the goal of mimicking the *in vivo* microenvironment of the infection site. Although most of these models were initially developed with the aim of understanding cell differentiation and tumorigenesis and as substitutes for animal tests, in recent years they have been increasingly adapted for other purposes, such as infection research. 3D tissue models based on human cells offer multiple advantages over 2D cell culture and animal models, particularly when the pathogen shows human host specificity.

This Research Topic aims to introduce the recent advances in the field of application and development of 3D tissue models in infection research. Two review articles offer overviews of the models for the urinary tract and gonococcal infections with an emphasis on the 3D tissue models. In addition, five original research papers applied various 3D tissue models to address timely and important research questions.

“Recurrent urinary tract infection: a mystery in search of better model systems” by Murray et al. offers a comprehensive overview of the disease complexity and various models used in the investigation of urinary tract infections. The review article also provides an in-depth discussion on the potential of currently available models for improvement. The proposed improvements aim to recapitulate key features of the human bladder, such as tissue architecture and stretchability, and exposure of the tissue to urine and liquid flow. The review article “Tissue models for *Neisseria gonorrhoeae* research - from 2D to 3D” by Heydarian et al. focuses on the causative agent of gonorrhea and the models used for studying gonococcal infection. Apart from providing a detailed summary of the existing animal and *in vitro* models, this review particularly focuses on newly developed 3D tissue models that could be applied for the research of the various aspects of gonococcal infection.

The original research papers in this collection introduce various 3D tissue models, including skin, tracheobronchial and nasal mucosa, colon, and human corneal, and the applications of these models to study infections with *Staphylococcus aureus*, *Bordetella pertussis*, and *Salmonella* Typhimurium.

In the “An *in vitro* mixed infection model with commensal and pathogenic staphylococci for the exploration of interspecific interactions and their impacts on skin physiology” paper, Kohda et al. utilized LabCyte EPI-MODEL, a commercially available 3D skin model based on foreskin epidermal keratinocytes attached to an artificial membrane by a layer of nutrient agar, to study *S. aureus* infection. Their data show that the presence of *Staphylococcus epidermidis*, a commensal bacterium, attenuates the cytotoxicity and cytokine production induced by pathogenic *S. aureus* and reduces *S. aureus* invasion into deeper tissue layers.

Two research papers investigated *B. pertussis* infection using models generated by seeding primary human fibroblasts/epithelial cells of the human airway mucosa on the collagen scaffold derived from the porcine small intestine (the so-called SIS scaffold). Whereas the paper “Activity of tracheal cytotoxin of *Bordetella pertussis* in a human tracheobronchial 3D tissue model” by Kessie et al. investigated the adverse effects of tracheal cytotoxin and lipopolysaccharide on 3D models of human tracheobronchial mucosa, the paper “Susceptibility of human airway tissue models derived from different anatomical sites to *Bordetella pertussis* and its virulence factor adenylate cyclase toxin” by Sivarajan et al. showed that the nasal tissue 3D models react differently than the models of the tracheobronchial tissue to the adenylate cyclase toxin of *B. pertussis*. Both papers introduce SIS-scaffold-based models of the respiratory tract tissues as an effective substitute for animal-derived models, such as hamster tracheal explants.

Distinct from the models used in the above three studies, where host cells were grown on flat support in a Transwell^®^-like system to form the apical and the basal part of the model, the study by Barilla et al., “Spaceflight analogue culture enhances the host-pathogen interaction between *Salmonella* and a 3-D biomimetic intestinal co-culture model” introduces a colon model combined with macrophages on microcarrier beads in rotating wall vessel bioreactor. This model was used to investigate the effect of physical forces caused by spaceflight on the colonization of colonic epithelium/macrophage models by *Salmonella* Typhimurium.

Finally, the research paper “Key role of staphylococcal fibronectin binding proteins during the initial stage of *Staphylococcus aureus* keratitis in humans” by Maurin et al. presents a human corneal explant model cultivated in a so-called active storage machine developed by the authors, which can support long-term culture and regeneration of the multilayered corneal epithelium. This model was applied for studying the early stage of *S. aureus*-induced keratitis. The authors demonstrate that epithelial injury and exposure of the underlying unmasked fibronectin molecules are required for *S. aureus* attachment and internalization.

The variety of 3D models and their applications presented in this collection of research and review articles clearly shows the great need for suitable models for the infection research field. Such need will continue to drive the development of novel 3D tissue models and the adaptation and improvement of the existing ones. We hope that this Research Topic will be an interesting introduction for you as readers to this field and provide you with a source of information and inspiration for the future.

## Author contributions

VK-P wrote the manuscript. WS, SB, and MM contributed with comments and suggestions. All authors contributed to the article and approved the submitted version.

## Conflict of interest

The authors declare that the research was conducted in the absence of any commercial or financial relationships that could be construed as a potential conflict of interest.

## Publisher’s note

All claims expressed in this article are solely those of the authors and do not necessarily represent those of their affiliated organizations, or those of the publisher, the editors and the reviewers. Any product that may be evaluated in this article, or claim that may be made by its manufacturer, is not guaranteed or endorsed by the publisher.

